# Supplementary material for: Signalling adjustments to direct and indirect environmental effects on signal perception in meerkats
Source: PLoS One. 2020 Aug 27;15(8):e0238313. doi: 10.1371/journal.pone.0238313 (PMC7451564; doi:10.1371/journal.pone.0238313)
Supplement: S1 File — (DOCX) [file pone.0238313.s001.docx]

**Supplementary material for** **“Signalling adjustment and its limitations to challenging environmental conditions in meerkats”**

**Supplement 1: Additional information on results of the models fitted for the different analysis.**

**S1 Table.** Model summary statistics comparing close call rate between the different environmental conditions, the distance to the nearest neighbour (Distance) and the presence of pups. The close call rate was used as response variable in a GLMM with a Poisson error distribution.

| Explanatory Variables | Estimate | Confidence intervals  [0.025, 0.975] | SE | z value | P value |
| --- | --- | --- | --- | --- | --- |
| Intercept | 1.11 | [0.60, 1.63] | 0.26 | 4.23 | <0.001 |
| **Distance: 0-5 m** | **0.28** | **[0.12, 0.44]** | **0.08** | **3.44** | **<0.001** |
| Env.: Dry | -0.13 | [-0.71, 0.45] | 0.30 | -0.78 | 0.653 |
| **Env.: Drought** | **0.69** | **[0.12, 1.26]** | **0.29** | **2.38** | **0.017** |
| Pups present | -0.42 | [-0.85, 0.01] | 0.22 | -1.91 | 0.056 |
| 0-5m * Dry | -0.08 | [-0.29, -0.13] | 0.11 | -0.78 | 0.439 |
| **0-5m * Drought** | **-0.22** | **[-0.39, -0.04]** | **0.09** | **-2.46** | **0.014** |

*Significant results are marked in bold

**S2 Table.** Post-hoc pairwise comparisons of the effect of the interaction between environmental conditions (Env.) and distance to the nearest neighbour (Distance) on close call rate. The comparisons were performed post-hoc from a GLMM with a Poisson error distribution

| Paired contrasts | Estimate | Confidence intervals  [0.025, 0.975] | SE | z value | P value |
| --- | --- | --- | --- | --- | --- |
| **Env.: Wet * Distance: 0-5m – Env.: Wet * Distance: >5m** | **0.28** | **[0.06, 0.50]** | **0.08** | **3.44** | **0.005** |
| **Env.: Dry * Distance: 0-5m – Env.: Dry * Distance: >5m** | **0.20** | **[0.02, 0.38]** | **0.07** | **2.96** | **0.022** |
| Env.: Drought * Distance: 0-5m – Env.: Drought * Distance: >5m | 0.06 | [-0.02, 1.54] | 0.03 | 1.85 | 0.340 |
| Env.: Wet * Distance: 0-5m – Env.: Dry * Distance: 0-5m | -0.22 | [-0.97, 0.53] | 0.28 | -0.77 | 0.958 |
| Env.: Wet * Distance: 0-5m – Env.: Drought * Distance: 0-5m | 0.47 | [-0.28, 1.23] | 0.28 | 1.69 | 0.444 |
| **Env.: Dry * Distance: 0-5m – Env.: Drought * Distance: 0-5m** | **0.69** | **[0.09, 1.29]** | **0.22** | **3.09** | **0.015** |
| Env.: Wet * Distance: >5m – Env.: Dry * Distance: >5m | -0.13 | [-0.93, 0.66] | 0.30 | -0.45 | 0.996 |
| Env.: Wet * Distance: >5m – Env.: Drought * Distance: >5m | 0.69 | [-0.09, 1.48] | 0.29 | 2.38 | 0.114 |
| **Env.: Dry * Distance: >5m – Env.: Drought * Distance: >5m** | **0.83** | **[0.20, 1.45]** | **0.23** | **3.54** | **0.003** |

*Significant results are marked in bold

**S3 Table.** Results of the Dirichlet model, comparing the proportion of time an individual spent within a specific distance category to its nearest neighbour between the different environmental conditions.

| Distance category | Explanatory variable | Estimate | Confidence interval [2.5%, 97.5%] | Std. error | z value | p value |
| --- | --- | --- | --- | --- | --- | --- |
| 0-2 m | Intercept | 0.47 | [0.15, 0.79] | 0.16 | 2.90 | <0.001 |
|  | **Env.: Dry** | **-0.39** | **[-0.76, -0.03]** | **0.19** | **-2.09** | **0.036** |
|  | **Env.: Drought** | **-0.65** | **[-1.01, -0.28]** | **0.19** | **-3.47** | **<0.001** |
| 2-5 m | Intercept | -0.96 | [-1.23, -0.68] | 0.14 | -6.72 | <0.001 |
|  | Env.: Dry | 0.24 | [-0.09, -0.57] | 0.17 | 1.45 | 0.147 |
|  | **Env.: Drought** | **0.51** | **[0.18, 0.83]** | **0.17** | **3.02** | **0.003** |
| 5-10 m | Intercept | -1.42 | [-1.70, -1.15] | 0.14 | -10.16 | <0.001 |
|  | Env.: Dry | -0.21 | [-0.52, -0.11] | 0.16 | -1.27 | 0.205 |
|  | Env.: Drought | -0.02 | [-0.34, 0.30] | 0.16 | -0.12 | 0.904 |
| > 10 m | Intercept | -1.69 | [-1.96, -1.41] | 0.14 | -12.09 | <0.001 |
|  | Env.: Dry | -0.13 | [-0.45, 0.19] | 0.16 | -0.81 | 0.418 |
|  | Env.: Drought | -0.10 | [-0.42, 0.22] | 0.16 | -0.63 | 0.532 |

*Significant results are marked in bold

**S4 Table.** Model summary statistics for the probability of group splits between wet, dry and drought conditions and depending on group size. The number of group splits was used as response variable in a GLMM with binomial error distribution.

| Explanatory Variables | Estimate | Confidence intervals  [0.025, 0.975] | SE | z value | P value |
| --- | --- | --- | --- | --- | --- |
| Intercept | -6.20 | [-7.04, -5.36] | 0.43 | -14.54 | < 0.001 |
| **Env.: Dry** | **-0.79** | **[-1.24, -0.36]** | **0.23** | **-3.54** | **< 0.001** |
| Env.: Drought | 0.36 | [-0.10, 0.82] | 0.24 | 1.52 | 0.129 |
| **Group size** | **0.11** | **[0.08, 0.14]** | **0.02** | **7.23** | **< 0.001** |

*Significant results are marked in bold

**S5 Table.** Post-hoc pairwise comparisons of the effect of environmental conditions (Env.) on the probability of group split. The comparisons were performed post-hoc from a GLMM with a binomial error distribution

| Paired contrasts | Estimate | Confidence intervals  [0.025, 0.975] | SE | z value | P value |
| --- | --- | --- | --- | --- | --- |
| **Env.: Wet – Env.: Dry** | **-0.80** | **[-1.33, -0.27]** | **0.23** | **-3.54** | **0.001** |
| Env.: Wet – Env.: Drought | 0.36 | [-0.20, 0.91] | 0.24 | 1.52 | 0.283 |
| **Env.: Dry – Env.: Drought** | **1.16** | **[0.61, 1.70]** | **0.23** | **4.98** | **< 0.001** |

*Significant results are marked in bold

**S6 Table.** Model summary statistics from a linear mixed-effects model (LMM) comparing morning weight (as a proxy for body condition) between wet, dry and drought conditions (Env.).

| Explanatory Variables | Estimate | Confidence intervals  [0.025, 0.975] | SE | DF | t value | P value |
| --- | --- | --- | --- | --- | --- | --- |
| Intercept | 582.50 | [559.91, 605.09] | 11.53 | 6.21 | 50.54 | <0.001 |
| Env.: Dry | -22.19 | [-50.86, 6.47] | 14.62 | 4.10 | -1.52 | 0.202 |
| **Env.: Drought** | **-62.56** | **[-103.34, -21.79]** | **20.81** | **4.19** | **-3.01** | **0.037** |
| **Age** | **33.88** | **[31.55, 36.20]** | **1.19** | **669.03** | **28.58** | **<0.001** |

*Significant results are marked in bold

**S7 Table.** Post-hoc pairwise comparisons of the effect of environmental conditions (Env.) on morning weight. The comparisons were performed post-hoc from a LMM with a Gaussian error distribution

| Paired contrasts | Estimate | Confidence intervals  [0.025, 0.975] | SE | z value | P value |
| --- | --- | --- | --- | --- | --- |
| Env.: Wet – Env.: Dry | -22.2 | [-56.3, 11.9] | 14.6 | -1.52 | 0.278 |
| **Env.: Wet – Env.: Drought** | **-62.6** | **[-111, -14.0]** | **20.8** | **-3.01** | **0.007** |
| Env.: Dry – Env.: Drought | -40.4 | [-88.6, 7.90] | 20.7 | -1.95 | 0.122 |

*Significant results are marked in bold

**S8 Table.** Model summary statistics from a LMM comparing morning weight gain (as a proxy for foraging success) between wet, dry and extremely dry environmental conditions.

| Explanatory Variables | Estimate | Confidence intervals  [0.025, 0.975] | SE | DF | t value | P value |
| --- | --- | --- | --- | --- | --- | --- |
| Intercept | 6.05 | [4.95, 7.14] | 0.56 | 4.88 | 10.80 | <0.001 |
| Env.: dry | 1.22 | [-0.26, 2.70] | 0.75 | 4.03 | 1.62 | 0.181 |
| Env.: extreme | 0.32 | [-1.76, 2.41] | 1.06 | 4.00 | 0.30 | 0.777 |
| **Age** | **0.27** | **[0.19, 0.34]** | **0.04** | **645.10** | **7.05** | **<0.001** |

*Significant results are marked in bold

**S9 Table.** Post-hoc pairwise comparisons of the effect of environmental conditions (Env.) on morning weight gain (as a proxy for foraging success). The comparisons were performed post-hoc from a LMM with a Gaussian error distribution

| Paired contrasts | Estimate | Confidence intervals  [0.025, 0.975] | SE | z value | P value |
| --- | --- | --- | --- | --- | --- |
| Env.: Wet – Env.: Dry | 1.22 | [-0.54, 2.98] | 0.75 | 1.62 | 0.234 |
| Env.: Wet – Env.: Drought | 0.32 | [-2.16, 2.81] | 1.06 | 0.30 | 0.950 |
| Env.: Dry – Env.: Drought | -0.90 | [-3.37, 1.58] | 1.06 | -0.84 | 0.672 |

*Significant results are marked in bold

**S10 Table.** Model summary statistics from a GLMM with a Poisson error distribution comparing individual age between wet, dry and extremely dry environmental conditions. Note that all individuals were adults (> 1year).

| Explanatory Variables | Estimate | Confidence intervals  [0.025, 0.975] | SE | z value | P value |
| --- | --- | --- | --- | --- | --- |
| Intercept | 1.12 | [0.55, 1.66] | 0.28 | 3.89 | <0.001 |
| Env.: dry | 0.23 | [-0.51, 0.98] | 0.38 | 0.61 | 0.542 |
| Env.: extreme | 0.82 | [-0.17, 1.80] | 0.50 | 1.63 | 0.103 |

*Significant results are marked in bold

**S11 Table.** Post-hoc pairwise comparisons of the effect of environmental conditions (Env.) on individual age. The comparisons were performed post-hoc from a GLMM with a Poisson error distribution

| Paired contrasts | Estimate | Confidence intervals  [0.025, 0.975] | SE | z value | P value |
| --- | --- | --- | --- | --- | --- |
| Env.: Wet – Env.: Dry | 0.23 | [-0.65, 1.12] | 0.38 | 0.61 | 0.812 |
| Env.: Wet – Env.: Drought | 0.82 | [-0.35, 1.99] | 0.50 | 1.63 | 0.229 |
| Env.: Dry – Env.: Drought | 0.58 | [-0.67, 1.84] | 0.54 | 1.09 | 0.518 |

*Significant results are marked in bold


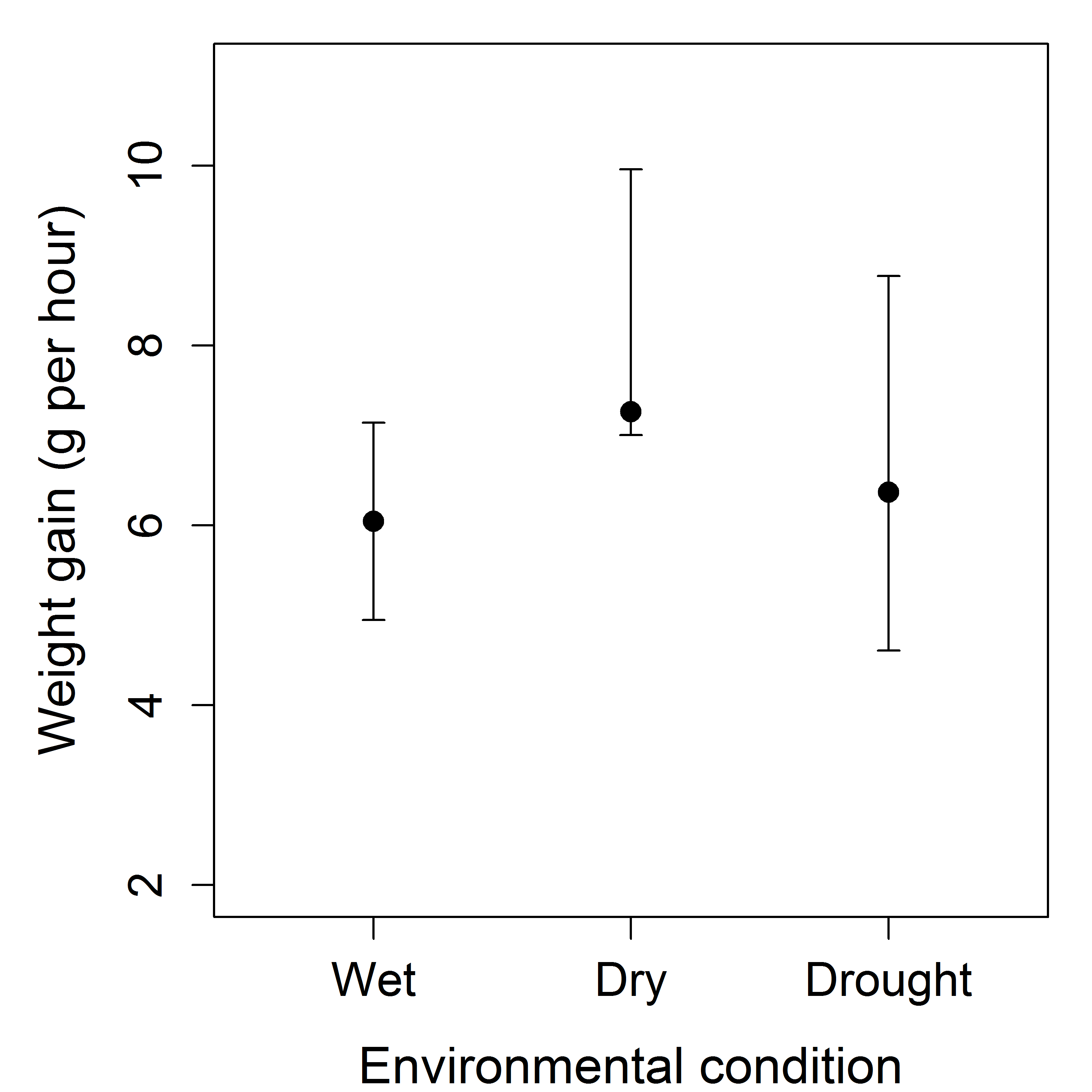


**S1 Fig.** (Generalised) Linear Mixed-effects Model estimates (points) and 95% confidence intervals (error bars) for mean weight gain of individuals (g/hour) under the different environmental conditions tested.
