# Supplementary material for: Signalling adjustments to direct and indirect environmental effects on signal perception in meerkats
Source: PLoS One. 2020 Aug 27;15(8):e0238313. doi: 10.1371/journal.pone.0238313 (PMC7451564; doi:10.1371/journal.pone.0238313)
Supplement: S2 File — (DOCX) [file pone.0238313.s002.docx]

**Supplementary material for** **“Signalling adjustment and its limitations to challenging environmental conditions in meerkats”**

**Supplement 2: Additional analysis on change in group speed with regards to the different environmental conditions**

Scarcely distributed resources due to reduced precipitation, can lead to a higher travel speed and longer movement distances for each group member and the whole group^[[1]](#footnote-1)^, which in turn leads to individuals moving faster out of one another’s perception range. Accordingly, we analysed long-term data to explore whether the travel speed of groups increased during drought conditions compared to dry and wet conditions. Observers following a meerkat group took a GPS (global positioning system) fix from the centre of the group every 15 min (accuracy: 95% of fixes within 5 m; eTrex 10, Garmin International Inc., Olathe, KS, USA). GPS fixes were taken in the morning from when the group started foraging until the end of the observation session (about 3 h after the start of foraging), and in the evening from the start of the session until the group reached its sleeping burrow (about 1.5 h after the start of the session). We calculated the distance a group travelled between two consecutive GPS fixes and the daily average distance per hour of a group for every day the groups had been visited during the different summer periods.

To test whether meerkat groups increased their movement distance per minute with increasing dryness, we square root transformed the mean daily speed of a group and fitted a Linear mixed effects mode with environmental condition and group size as explanatory variable and summer period and group identity as random terms. After stepwise model reduction using the AIC we removed group size as explanatory variable to improve the model fit.

Based on GPS fixes collected at 15 min intervals at the centre of a foraging group, meerkats travelled slowest under wet conditions at an average of 4.38 m/min, while they travelled on average with 4.39 m/min under dry conditions and fastest, at on average 4.42 m/min, under the drought condition (LRT, square root transformed speed: χ² = 9.53, P value < 0.001, Figure S2, Table S8). Although, as predicted, we found a gradual increase in group-travel speed with increasing dryness, this effect was very small and probably does not lead to any meaningful differences in perception range and thus in the calling behaviour of individual meerkats.

**S2 Table.** Model summary statistics from a LMM comparing mean daily group speed between wet, dry and drought conditions.

| Explanatory Variables | Estimate | Confidence intervals  [0.025, 0.975] | SE | DF | t value | P value |
| --- | --- | --- | --- | --- | --- | --- |
| Intercept | 0.27 | [0.26, 0.28] | 0.004 | 6.85 | 63.52 | 0.030 |
| Env.: Dry | 0.01 | [0.00, 0.02] | 0.005 | 3.84 | 2.22 | 0.093 |
| **Env.: Drought** | **0.03** | **[0.01, 0.04]** | **0.007** | **3.89** | **3.49** | **0.026** |

*Significant results are marked in bold


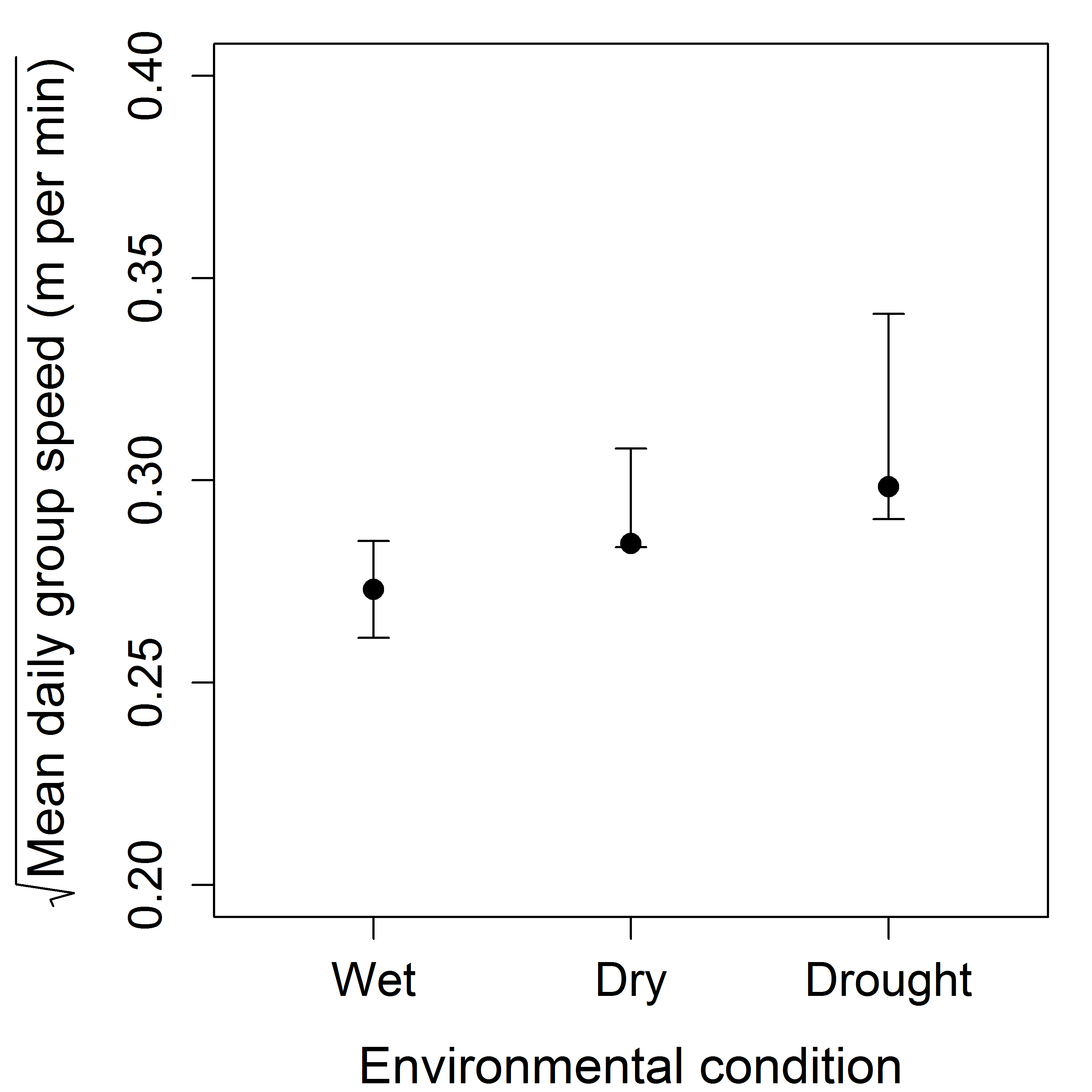


**S2 Fig**. (Generalised) Linear Mixed-effects Model estimates (points) and 95% confidence intervals (error bars) for mean daily group speed (m/min) under the different environmental conditions tested.

1. Henzi SP, Byrne RW, Whiten A. 1992. Patterns of movement by baboons in the Drakensberg mountains: Primary responses to the environment. Int J Primatol. 13(6):601–629. doi:10.1007/BF02551256 [↑](#footnote-ref-1)
